# Leukocytes and Neutrophil–Lymphocyte Ratio as Indicators of Insulin Resistance in Overweight/Obese School-Children

**DOI:** 10.3389/fnut.2021.811081

**Published:** 2022-05-06

**Authors:** Elena Rodríguez-Rodríguez, M. Dolores Salas-González, Rosa M. Ortega, Ana M. López-Sobaler

**Affiliations:** ^1^VALORNUT Research Group, Analytical Chemistry Unit, Department of Chemistry in Pharmaceutical Science, Faculty of Pharmacy, Complutense University of Madrid, Madrid, Spain; ^2^VALORNUT Research Group, Department of Nutrition and Food Science, Faculty of Pharmacy, Complutense University of Madrid, Madrid, Spain

**Keywords:** inflammation, insulin resistance, overweight, obesity, children

## Abstract

**Background and Aims:**

Inflammation in overweight/obesity may condition the development of insulin resistance (IR). New markers of inflammation and systemic inflammation, such as leukocyte and platelet counts, the neutrophil-to-lymphocyte ratio (NLR), platelet-to-lymphocyte ratio (PLR), and monocyte-to-lymphocyte ratio (MLR), could be used as indicators of IR. The aim of the present study was to investigate the relationship between these markers and IR in overweight/obese children.

**Materials and Methods:**

A total of 1,035 schoolchildren were studied (398 overweight/obese). Anthropometric, hematological and biochemical measurements were collected. Inflammation was considered to be present when the values of leukocytes, platelets, NLR, PLR and MLR were ≥*p*75 for each sex. IR was defined as HOMA-IR >3.16.

**Results:**

In overweight/obese schoolchildren, leukocytes were higher in both boys and girls, and platelets and NLR were higher in boys with IR than in boys with insulin sensitivity. An association between leucocytes and NLR values (≥*p*75) and IR was found in the entire population [2.911 (1.328–6.381); *p* = 0.008 and 2.660 (1.185–5.968); *p* = 0.018, respectively] and in boys [9.255 (1.896–45.168); *p* = 0.006 and 6.996 (1.377–35.544); *p* = 0.019, respectively].

**Conclusion:**

Elevated white blood cell values and elevated NLR in overweight/obese children, and particularly in boys, could be indicators of the presence of IR in these subjects and should be considered when predicting possible complications, such as type II diabetes mellitus.

## Introduction

Obesity is becoming a global epidemic, increasing the health burden of associated complications of insulin resistance and diseases such as cardiovascular disease (1) or type II diabetes mellitus (2). One of the major causes linking the development of insulin resistance (IR) to obesity is the inflammatory state that occurs in obesity (3–7). Briefly, inflammatory mediators bound to their membrane receptor activate protein kinases, which in turn inhibit insulin receptor substrate (IRS), reducing insulin signaling and leading to reduced translocation of glucose transporter 4 (GLUT4) from the cytosol to the cell membrane, thus decreasing blood glucose uptake. As a compensatory response, insulin hypersecretion occurs, which explains the typical hyperinsulinemia that can occur in obese people (8).

Among the main and most studied inflammatory mediators released by adipose tissue are interleukin-6 (IL-6), tumor necrosis factor alpha (TNF-α), angiotensinogen and the growth factor TGF-beta, and hormones secreted by adipocytes, such as leptin and resistin. Along with adipose tissue, acute-phase proteins of hepatic origin, C-reactive protein (CRP), amyloid A, fibrinogen and plasminogen activation inhibitor have also been linked to the development of inflammatory processes (9). In particular, obesity and insulin resistance have been shown to be positively associated with increased levels of CRP and cytokines in numerous studies (10). For example, it has been observed that increased serum TNF- α and IL-6 levels but decreased levels of adiponectin and IL-10 are associated with increased inflammation, tissue injury and complications of obesity (11). Similarly, high levels of IL-6 and IL-15 have been detected in the salivary samples of children with obesity (12). However, they are expensive markers, and thus easily available and low-cost indicators are desirable.

Complete blood count is an inexpensive and simple laboratory examination, and in recent years, leukocyte and platelet counts, neutrophil-to-lymphocyte ratio (NLR), platelet-to-lymphocyte ratio (PLR), and monocyte-to-lymphocyte ratio (MLR) have been studied as inflammatory markers in various chronic subclinical diseases (13–17). These markers are easily measurable and available (they can be calculated from routine complete blood count), cost-effective and reliable, and can therefore be used as an index of severity of the immune response (18).

However, there are few studies, especially in school children, using these indicators of inflammation. Therefore, based on the findings of previous studies, the aim of the present study was to investigate the relationship between these novel indicators of systemic inflammation and IR in overweight/obese (OW/OB) children.

## Materials and Methods

### Subjects

A convenience sample of 1,035 schoolchildren between 8 and 13 years of age were studied. To select them, the head teachers of different primary schools in 5 Spanish provinces (Madrid, Barcelona, Valencia, A Coruña and Sevilla) were contacted by telephone, and the characteristics and importance of the intended study were explained to them. Once the acceptance of the head teacher and the parents' association had been obtained, a meeting was held with the families of the schoolchildren in which the details of the study were explained to them, their questions were answered, and their signed authorization was requested. All participants took part in the study on a voluntary basis.

Inclusion criteria were:

- They were in 4, 5, and 6^th^ grade of primary school and between 8 and 13 years of age.
- Written informed consent signed by the child's parents and/or guardians.
- Absence of diseases related to the metabolism or intake of nutrients, such as metabolic or endocrine diseases.
- Not having a pharmacological treatment that interferes with intake, bioavailability or implies changes in nutritional needs.

Exclusion criteria were:

- Lack of signed authorization to take part in the study.
- Failure to accept some of the conditions required to take part in the study.
- Not attending school on the days on which the tests and interviews were carried out.
- Having any pathology (e.g., endocrine, metabolic, inadequate renal function) which, due to its characteristics or severity, could contribute to modifying eating habits (and nutrient intake).

The acceptance rate was roughly calculated. Having in mind the line of each school (number of classes in each course: 4°, 5° of 6°) and that the ratio of each class is usually 25, the number of potential students to be studied were 3,850. Therefore, since 1,035 students finally participated, the approximate participation rate was 27%.

This study was conducted in accordance with the guidelines established in the Declaration of Helsinki, and all procedures involving human subjects were approved by the Human Research Review Committee of the Faculty of Pharmacy of the Complutense University of Madrid.

### Anthropometric Study

All measurements (weight, height, waist, triceps skinfold thickness and biceps skinfold thickness) were taken in the morning in the schools themselves and according to the standards established by the World Health Organization (19).

Weight and height were measured using a digital electronic scale (model SECA ALPHA, GMBH & Co., Igni, France) (range: 0.1–150 kg, accuracy 100 g) and a Harpenden digital stadiometer (Pfifter, Carlstadt, N.J., USA) (range: 70–205 cm, accuracy 1 mm), respectively. Measurements were taken with the schoolchildren in their underwear. Body mass index (BMI) was calculated by dividing weight (kg) by height squared (m^2^).

Overweight and obesity were defined in terms of body mass index (BMI) using the reference tables of Hernández et al. (20), i.e., overweight as BMI ≥85th percentile, and obesity as BMI ≥97th percentile. According to this criterion, 398 had OW/OB (218 boys and 180 girls).

The plane of the waist was taken as the point midway between the inferior margin of the last rib and the crest of the ilium. Waist measurements were determined in triplicate using Holtain flexible metallic tape (range 0–150 cm, precision 1 mm) held snugly around the body (although not tight enough to compress the subcutaneous adipose tissue) and with the subject standing relaxed. An assistant helped to hold the tape on the side of the subject's body opposite to the measurer. The mean of the three measurements was used for analysis.

The waist-to-height ratio (WH ratio) was calculated as an indicator of the presence of central obesity (21).

Triceps and biceps skinfold thickness was measured on the right side in triplicate to the nearest millimeter using a Holtain skinfold caliper (Holtain Ltd, Crymych, Wales). All calipers were calibrated each day prior to taking measurements.

The percentage body fat (%BF) was determined using the equation of Parizkova (22):

girls: %BF = 39.032Y - 30.084
boys: %BF = 32.914Y - 21.973
where Y= log (sum of skin fold thickness of the biceps + triceps)

### Physical Activity

An adapted physical activity daily questionnaire (23) which has been previously used in other studies (24–28), was filled out by the parents about their children. Information about the length of time spent sleeping, eating and playing sports was recorded. An activity coefficient was established for each subject by multiplying the time spent in each activity by established coefficients (29, 30), i.e., 1 for sleeping and resting, 1.5 for very light activities (those that can be performed sitting or standing up, such as studying, doing homework, or painting), 2.5 for light activities (e.g., walking), 5 for moderate activities (e.g., playing tennis, skiing, dancing), and 7 for intensive activities (e.g., playing basketball) - and then dividing by 24 h.

### Hematological and Biochemical Studies

Blood samples were taken by venipuncture after 12 h of fasting, between 8 and 9 a.m.

The complete blood cell counts and differential counts of white blood cells in anticoagulated whole blood were determined by a Model S Coulter Counter (Coulter Electronic Limited, Luton, UK). Inflammation was considered to be present when platelet and leukocyte values were ≥*p*75 for each sex, i.e., for platelets, 335.25 × 10^3^/μL in boys and 334 × 10^3^/μL in girls and for leukocytes, 7.685 × 10^3^/μL in boys and 7.3 × 10^3^/μL in girls.

Neutrophil-to-lymphocyte ratio (NLR) dividing absolute neutrophil count by absolute lymphocyte count, monocyte-to-lymphocyte ratio (MLR) dividing the absolute monocyte count by the absolute lymphocyte count, and platelet-to-lymphocyte ratio (PLR) dividing the platelet count by the lymphocytes were calculated. Inflammation was considered to exist for these ratios ≥*p*75 according to sex (1.487 and 1.424 for NLR, 0.222 and 0.234 for MLR and 137.884 and 139.71 for PLR in girls and boys, respectively).

Serum high-sensitivity C-reactive protein (hs-CRP) was tested by immunonephelometry (CardioPhase^®^ hsCRP assay; Dade Behring, Milan, Italy) (CV = 3.1%). Inflammation was considered to be present when the hs-CRP value was ≥*p*75 according to sex (girls: 0.09 mg/dL and boys: 0.1 mg/dL).

Fasting insulin was determined by chemiluminescence (Abbott Diagnostics Division, Spain). Glucose levels were assessed by colorimetric techniques using the glucose oxidase-peroxidase method (Vitros GLU slides, Rochester, New York, USA).

The HOMA index was calculated from glucose and insulin values to determine the degree of existing insulin resistance in schoolchildren (31, 32) using the following formula:

HOMA = fasting plasma glucose (mmol/L) × fasting serum insulin (mU/L)/22.5.

The cutoff point for HOMA to identify individuals with insulin resistance was 3.16, which has been used in other studies for obese pediatric populations (33, 34).

From the total population, we obtained insulin values of 895 schoolchildren, glucose of 962 (HOMA could be calculated as 890), hs-CRP of 555 and complete blood count of 996.

### Statistical Analysis

Data was tested for normality distribution according to the sex using Kolmogorov Smirnov Test. All continuous variables were not normally distributed. The median and interquartile range (*p*25–*p*75) were calculated for all variables. Differences between means were established using the Mann–Whitney test, as the variables did not follow a normal distribution. Pearson's logistic and Spearman's monotonic correlation coefficients between data were also calculated. A univariate analysis was first performed to identify any potential predictor variables. Variables with a *p*-value < 0.2 according to a univariate analysis and those considered to be clinically relevant were included in the multivariate analysis to determine any independent predictors of the primary outcome variables. Also, those variables known or likely to be associated with insulin resistance were included in the logistic regression models as potential independent variables were examined in the multivariate model using logistic regression. To avoid multicollinearity, only one variable was retained in a set of variables with a significative correlation. SPSS version 25 for Mac was used to process and analyze all data.

## Results

Boys and girls with normal and low weight had lower values for BMI, waist/height, body fat percentage, platelets, insulin, HOMA and hs-CRP than those with OW/OB. In addition, glucose in girls and leucocytes in boys with low/normal weight were lower than the values found in the group with OW/OB. When comparing the parameters studied by sex within each group (normal and underweight or overweight/obese), we observed that within the OW/OB group, girls had higher, z-BMI, insulin and HOMA-IR values than boys. Insulin and HOMA-IR parameters were also different between sexes in the underweight/normal weight group (Table 1).

**Table 1:** Anthropometric, blood and biochemical parameters in the schoolchildren studied according to sex and weight status.

	**Under and normal weight**		**Overweight/obesity**	
**Variable**	**Boys**	**Girls**	**Boys**	**Girls**
BMI (kg/m^2^)	16.91 (15.84–17.98)a^*^	17.17 (15.99–18.52)a	21.48 (20.01–23.62)	22.06 (20.52–23.45)
z-height	–0.78 (–1.01 to –0.18)a^*^	–0.60 (–0.94 to –0.02)a	0.61 (0.10–1.18)	0.82 (0.18–1.40)
z-BMI	0.03 (–0.47 to 0.61)a	0.10 (–0.49 to 0.62)a	1.90 (1.42–2.32)^**^	1.59 (1.36–1.98)
WH ratio	0.43 (0.42–0.45)a^*^	0.43 (0.41–0.45)a	0.50 (0.48–0.55)	0.50 (0.47–0.54)
Body fat (%)	15.64 (12.56–19.52)a^***^	20.44 (16.18–24.46)a	26.88 (23.50–30.47)^**^	29.72 (26.31–32.00)
Activity coefficient	1.53 (1.45–1.61)	1.51 (1.43–1.60)	1.51 (1.44–1.61)	1.50 (1.43–1.58)
Leucocytes (10^3^/μL)	6.40 (5.40–7.62)a	6.30 (5.50–7.30)	6.80 (5.73–7.75)	6.44 (5.50–7.65)
Platelets (10^3^/μL)	289 (251–329)a	285 (254–327)a	302 (265–343)	304 (263–347)
NLR	1.12 (0.85–1.38)	1.11 (0.85–1.48)	1.09 (0.87–1.49)	1.15 (0.94–1.50)
MLR	0.18 (0.15–0.23)	0.18 (0.15–0.22)	0.19 (0.15–0.24)	0.18 (0.14–0.23)
PLR	111.74 (91.02–136.15)	112.87 (94.06–136.65)	116.69 (92.62–141.48)	118.03 (94.59–146.29)
Glucose (μg/dL)	85.00 (80.00–90.00)^**^	83.00 (77.00–89.00)a	87.00 (81.00–91.00)	85.00 (81.00–90.00)
Insulin (μU/mL)	3.78 (2.24–5.39)a^***^	5.09 (3.24–7.58)a	6.14 (4.32–8.95)^***^	8.27 (5.65–12.50)
HOMA-IR	0.78 (0.47–1.14)a^***^	1.07 (0.67–1.58)a	1.32 (0.88–1.98)^***^	1.80 (1.16–2.62)
hs–CRP (mg/dL)	0.05 (0.05–0.06)a	0.05 (0.04–0.07)a	0.07 (0.05–0.19)	0.08 (0.05–0.18)

WH ratio: weight to height ratio. a: Differences between the same sex comparing normal/underweight with overweight/obesity. Differences between the sexes within the group of normal/underweight or overweight/obesity are shown with asterisks (*p < 0.05; **p < 0.01; ***p < 0.001).

Leucocyte levels correlated with body fat percentage and waist-to-height ratio in boys with OW/OB (Rho = 0.271, *p* = 0.005 and Rho = 0.179, *p* = 0.011, respectively). This relationship was not seen in girls with OW/OB or in either sex in the underweight/normal weight group. Platelet concentration correlated with BMI (Rho = 0.154; *p* = 0.029) and waist-to-height ratio (Rho = 0.183; *p* = 0.010) in boys and only with waist-to-height ratio (Rho = 0.293; *p* = 0.010) in girls.

**Figure 1:**
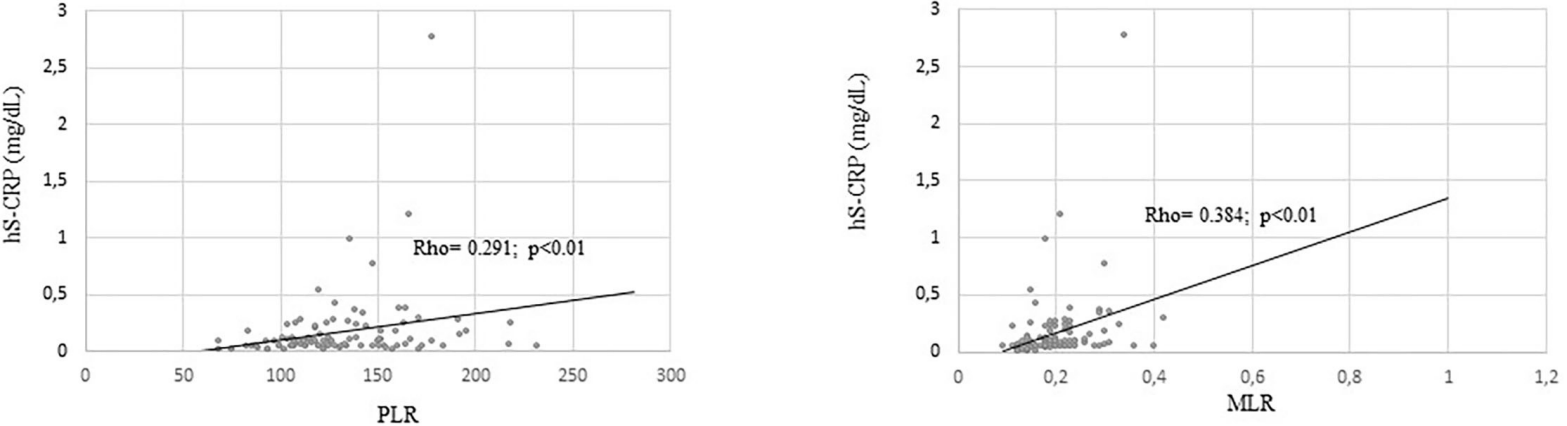
Significative correlation coefficients between hs-CRP (mg/dL) and inflammatory markers in OW/OB girls.

**Figure 2:**
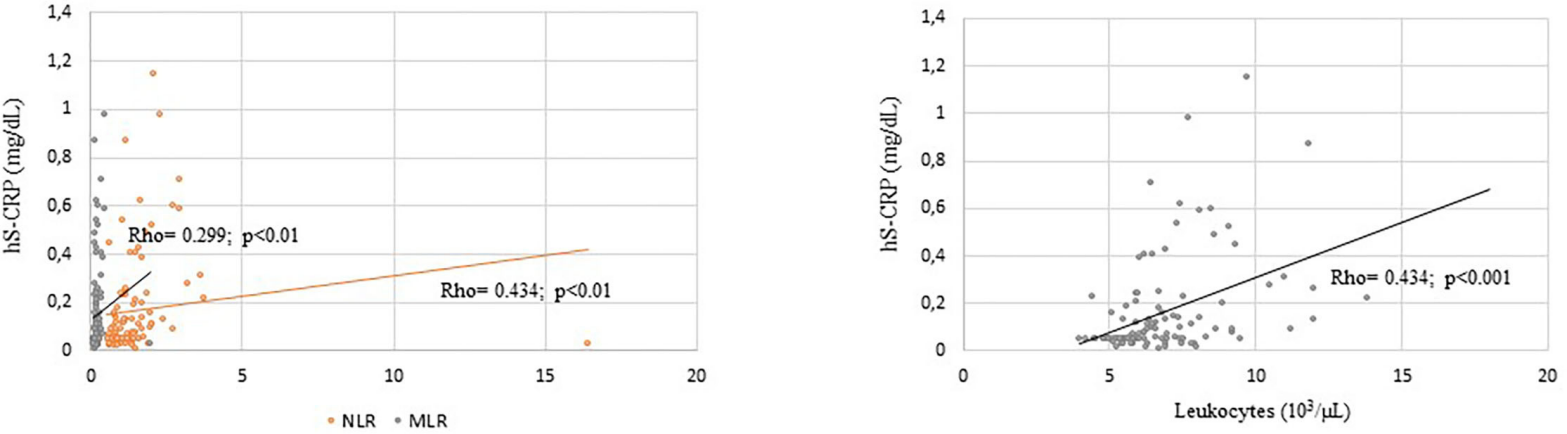
Significative correlation coefficients between hs-CRP (mg/dL) and inflammatory markers in OW/OB boys.

When overweight/obese schoolchildren were considered, hs-CRP concentration was related to leukocyte concentration and NLR in boys, to MLR in both boys and girls and to PLR ratio in girls (Figures 1, 2). No correlation was found between hs-CRP and platelet concentration (Rho = 0.107) and PLR (Rho = 0.131) in boys or between hs-CRP and platelet concentration (Rho = 0.178), leukocyte concentration (Rho = 0.008) and NLR (Rho = 0.177) in girls.

Regarding hematological and biochemical parameters related to inflammation in OW/OB schoolchildren, leukocytes, platelets and NLR were higher in boys with insulin resistance, as measured by the HOMA-IR index, than in those with insulin sensitivity. All other markers showed no statistically significant differences. In the case of girls with OW/OB, those with IR did not have higher values of any marker of inflammation than those without IR, except for leukocytes, which were higher in the former (Table 2).

**Table 2:** Anthropometric, hematological and blood parameters in schoolchildren with overweight/obesity according to HOMA-IR value and sex.

	**Boys**		**Girls**	
	**HOMA-IR>3.16**	**HOMA-IR <3.16**	**HOMA-IR>3.16**	**HOMA-IR <3.16**
	***n* = 11**	***n* = 171**	***n* = 22**	***n* = 133**
BMI (kg/m^2^)	24.52 (22.44–27.51)	21.26 (19.97–23.24)^**^	22.43 (20.81–23.63)	21.73 (20.36–23.17)
z-height	0.08 (–1.03 to 0.41)	0.04 (–0.43 to 0.61)	0.90 (–0.18 to 1.39)	0.18 (–0.43 to 0.79)
z-BMI	2.46 (2.01–2.87)	1.86 (1.40–2.24)^**^	1.60 (1.41–2.05)	1.56 (1.34–1.89)
WH ratio	0.57 (0.55–0.58)	0.50 (0.48–0.55)^***^	0.51 (0.47–0.53)	0.50 (0.47–0.53)
Activity coefficient	1.57 (1.48–1.67)	1.52 (1.44–1.62)	1.45 (1.42–1.51)	1.51 (1.43–1.61)^*^
Leucocytes (10^3^/μL)	8.64 (7.50–9.90)	6.80 (5.78–7.60)^***^	7.25 (6.00–8.25)	6.30 (5.40–7.50)^*^
Platelets (10^3^/μL)	356 (279–417)	300 (256.5–335)^*^	302.50 (277.50–358.25)	298 (250–333)
Glucose (mg/dL)	86.00 (80.00–92.00)	88.00 (82.00–91.00)	90.50 (79.75–95.00)	85.00 (81.00–89.50)^*^
Insulin (μU/mL)	17.50 (16.50–20.80)	5.91 (4.30–8.31)^***^	18.75 (16.45–20.88)	7.34 (5.30–10.75)^***^
HOMA–IR	3.93 (3.34–4.42)	1.24 (0.84–1.75)^***^	3.77 (3.37–5.00)	1.59 (1.07–2.19)^***^
NLR	1.50 (1.11–1.88)	1.07 (0.87–1.49)^*^	1.21 (0.99–1.60)	1.12 (0.91–1.46)
MLR	0.17 (0.14–0.25)	0.19 (0.15–0.24)	0.19 (0.14–0.28)	0.19 (0.14–0.23)
PLR	110.53 (93.62–134.40)	116.76 (90.66–141.18)	115.63 (96.78–140.91)	116.83 (92.68–142.76)
hs–CRP (mg/dL)	0.29 (0.06–0.83)	0.08 (0.05–0.16)	0.09 (0.05–0.24)	0.08 (0.05–0.18)

WH ratio, weight to height ratio. Differences within sex between absence or presence of IR are shown with asterisks (*p < 0.05; **p < 0.01; ***p < 0.001).

In the general sample and in boys with OW/OB, leukocyte and NLR values indicative of inflammation (≥*p*75) were found to be risk factors for insulin resistance (HOMA >3.16) (corrected for age, activity, z-BMI and central obesity) (Table 3).

**Table 3:** Association between high inflammatory indicators (≥75th of the total population) and the presence of IR in overweight/obese schoolchildren.

	**Total**		**Boys**		**Girls**	
	**OR (95% CI)**	***P*-value**	**OR (95% CI)**	**P-value**	**OR (95% CI)**	**P-value**
Leucocytes (10^3^/μL)	2.911 (1.328–6.381)	0.008	9.255 (1.896–45.168)	0.006	1.617 (0.591–4.426)	0.350
Platelets (10^3^/μL)	2.392 (1.056–5.419)	0.037	2.059 (0.470–9.016)	0.338	2.396 (0.844–6.797)	0.101
NLR	2.660 (1.185–5.968)	0.018	6.996 (1.377–35.544)	0.019	1.753 (0.602–5.109)	0.304
MLR	1.230 (0.505–2.993)	0.648	0.623 (0.110–3.522)	0.593	1.314 (0.441–3.919)	0.624
PLR	1.028 (0.427–2.475)	0.951	0.317 (0.039–2.608)	0.285	1.123 (0.371–3.400)	0.838
hs–CRP (mg/dL)	0.789 (0.234–2.660)	0.703	0.540 (0.076–3.816)	0.537	0.921 (0.173–4.905)	0.923

*Logistic regression analysis. Covariates: age, waist/height ratio, activity coefficient and z-BMI*.

## Discussion

Obesity represents the major risk factor for the development of insulin resistance during childhood and adolescence. The relationship between obesity and the existence of insulin resistance (and diabetes mellitus type II) has been well-described and studied previously in the literature (35). It has been estimated that ~55% of the variance in insulin sensitivity in children can be explained by total adiposity after adjusting for other confounders, such as age, sex, ethnicity, and pubertal stage (36). In the present study, when dividing the schoolchildren according to whether they were underweight/normal weight or OW/OB, as expected, the latter had higher levels of parameters related to weight status (BMI, WH ratio and body fat percentage) and those related to insulin resistance (insulin, HOMA-IR), as described in the literature (8). In addition, the parameters related to IR were higher in girls than in boys, as has also been seen in previous studies, which may be due to the hormonal influence that occurs at this age, with the onset of puberty (37).

Focusing on OW/OB, previous studies have shown that increased adiposity appears to increase leukocytes within the circulation and that OW children have higher levels of circulating leukocytes than normal weight children (38). In our study, leukocytes were higher in the OW/OB group than in the underweight/normal weight group only in boys, and a correlation was observed between leukocytes and some parameters indicative of obesity, such as fat percentage and waist/height ratio, in overweight/obese boys. In girls, these relationships were not observed, which could be due to differences in pubertal status between genders (which has not been taken into account), as it has been described as a potential factor contributing to leukocyte counts (39). In addition to total leukocyte counts, obesity has also been reported to increase platelet counts and platelet activation (40, 41), as well as CRP (42, 43), which could be related to the increase in IL-6 in these subjects, as this adipokine appears to induce hepatic CRP synthesis, as well as platelet and lymphocyte production (44, 45).

In our work, consistent with this, platelet and CRP values were higher in schoolchildren (both boys and girls) with OW/OB than in those with low/normal weight.

With regard to schoolchildren with OW/OB, who are at higher risk of developing IR and other complications, the major finding of the present study was that the entire population and boys with IR showed higher total leukocyte count and NLR than those without IR and that high values of these parameters (≥*p*75) were found to be risk factors for IR, suggesting the existence of an inflammatory response in these population and boys. In this regard, meta-analysis studies have shown that an increased count of circulating leukocytes is associated with a higher risk of type II diabetes mellitus (46). In addition, the association of all subtypes of circulating leukocytes with IR has been shown in high-risk individuals (47). Furthermore, our results coincide with other work in adults, such as that of Karakaya et al. (48), who, studying 96 patients with obesity and 40 healthy controls, found a positive correlation between insulin resistance and white blood cell counts and that the NLR was higher among obese patients with IR than among nonIR obese patients. These results suggest the presence of low-grade systemic inflammation in boys with OW/OB and IR, although hs-CRP, an acute inflammatory protein, showed no differences according to the presence or absence of IR. It is worth mentioning that, despite the above, an association between hs-CRP and total leukocytes and NLR was observed in boys, and thus these parameters could be used as markers similar to CRP in the determination of increased inflammation, as has also been observed in other studies (49, 50). For example, in an investigation of 225 Mexican children between 6 and 13 years old (106 obese and 119 normal-weight), an association was observed between CRP concentration and both leukocyte and platelet counts (51). Similarly, in another study in 13 patients with stable chronic obstructive pulmonary disease, there was a significant correlation of NLR with CRP (*r* = 0.309, *P* < 0.001) (52). It is worth mentioning that the association between NLR and hs-CRP was observed only in boys (whereas in girls, only PLR and hs-CRP were associated), which could be because these ratios, NLR and PLR levels, are heritable and influenced by sex, among other factors (53). Finally, it should be noted that the 95% CI for the leukocytes and the NLR presented in the multivariate analysis of boys are too wide. The width of the CI is highly dependent on the sample size, and studies with a large sample give a smaller confidence interval, which points to a high estimation precision with a high level of confidence. It would be desirable to conduct similar studies in obese children considering larger sample sizes.

Finally, some studies have shown that the number of platelets would be significantly higher in participants who were resistant to insulin than in those who were sensitive to insulin (54); although in our study we observed this difference in the boys studied, high platelet values (≥*p*75) did not constitute a risk factor for the appearance of IR in these children, nor was any relationship observed between the existence of IR and PLR. Relatedly, it has been reported that the NLR seems to predict inflammation better than the PLR because neutrophils play a dominant role in inflammation, as neutrophils may lead to increased endothelial permeability by releasing vasoactive and cytotoxic agents such as reactive oxygen species and digestive proteases during inflammation (55).

There are some limitations to the current study, such as the cross-sectional design of our work, that only hs-CRP was used to assess chronic inflammation and that a wider spectrum of inflammatory biomarkers, such as fibrinogen, IL-6, and TNF-α, would have been desirable. Furthermore, because it is a large community study, Tanner staging by a clinician was not possible and information about puberal status of the participant children was not included.

Accordingly, in view of our results, elevated leukocyte and NLR values in children, but particularly boys, with OW/OB could be indicators of the presence of insulin resistance in these subjects and should be considered when predicting/preventing possible complications, such as diabetes mellitus type II, in these boys.

## Data Availability Statement

The raw data supporting the conclusions of this article will be made available by the authors, without undue reservation.

## Ethics Statement

The studies involving human participants were reviewed and approved by Human Research Review Committee of the Faculty of Pharmacy of the Complutense University of Madrid. Written informed consent to participate in this study was provided by the participants' legal guardian/next of kin.

## Author Contributions

AL-S and RO contributed to conception and design of the study. MS-G organized the database. ER-R performed the statistical analysis and wrote the first draft of the manuscript. All authors contributed to the article and approved the submitted version.

## Funding

This work was supported by FISS project PI060318, INCERHPAN-UCM contract 210/2008 and a predoctoral contract financed by the Complutense University of Madrid and Banco Santander (CT63/19-CT64/19).

## Conflict of Interest

The authors declare that the research was conducted in the absence of any commercial or financial relationships that could be construed as a potential conflict of interest.

## Publisher's Note

All claims expressed in this article are solely those of the authors and do not necessarily represent those of their affiliated organizations, or those of the publisher, the editors and the reviewers. Any product that may be evaluated in this article, or claim that may be made by its manufacturer, is not guaranteed or endorsed by the publisher.

## Abbreviations

BF: body fat
BMI: body mass index
hs-CRP: high-sensitivity C-reactive protein
IR: insulin resistance
MLR: monocyte-to-lymphocyte ratio
NLR: neutrophil-to-lymphocyte ratio
PLR: platelet-to-lymphocyte ratio
OW: overweight
OB: obesity
WH: waist/height ratio.
